# Supplementary material for: Deletion of wfs1 Impairs Oligodendrocyte Precursor Cells Dorsal Distribution and Myelination Through the wfs1-hmgcs1 Axis in Zebrafish
Source: Biology (Basel). 2026 Aug 16;15(16):1402. doi: 10.3390/biology15161402 (PMC13510151; doi:10.3390/biology15161402)
Supplement: Supplementary file 1 [file biology-15-01402-s001.zip › File S1-Transcriptome sequencing data and differentially expressed genes (DEGs) in wfs1-- zebrafish at 5 dpf/2.KEGG/wfs1KOvsWT/ALL/src/dre05168.html]

dre05168


- dre:791704

- Up regulated genes

791704(1.64775824422649)

- dre:564295

- Down regulated genes

564295\_2(-1.27307321576804)

- dre:100149189

- Up regulated genes

100149189(1.21378518263532)

- dre:565338

- Down regulated genes

565338\_1(-2.75872437189811)
- dre:559564

- Down regulated genes

559564(-1.18025564761092)

- dre:368481

- Down regulated genes

368481(-2.13889418398421)

- dre:368481

- Down regulated genes

368481(-2.13889418398421)

- dre:406288

- Down regulated genes

406288\_1(-1.12731177210543)

- dre:58113

- Up regulated genes

58113(1.99980927955512)

- dre:30762

- Up regulated genes

30762(1.51549567324805)
- dre:368878

- Up regulated genes

368878(2.50701188401484)
- dre:30763

- Up regulated genes

30763(5.58822372755117)
- dre:571282

- Up regulated genes

571282(1.90251155561911)

- dre:768248

- Up regulated genes

768248(6.07490717007993)

- dre:561021

- Up regulated genes

561021(2.14284154145004)

- Down regulated genes

561021\_1(-4.68369619674599)

- dre:373107

- Down regulated genes

373107\_1(-7.07514976036473)

- dre:373107

- Down regulated genes

373107\_1(-7.07514976036473)

- dre:556826

- Up regulated genes

556826\_1(2.25777649220643)

- dre:30163

- Down regulated genes

30163(-1.56526278456924)

- dre:30400

- Down regulated genes

30400(-1.1592690100213)

- dre:100330617

- Up regulated genes

100330617\_1(1.54741913113035)

- dre:100000252

- Up regulated genes

100000252\_3(1.94434154854557) 100000252(1.09163460881879)

- Down regulated genes

100000252\_1(-1.7718817168434)

- dre:58100

- Down regulated genes

58100\_1(-4.1286149197908)

- dre:564266

- Up regulated genes

564266(2.42311476837666)

- dre:565338

- Down regulated genes

565338\_1(-2.75872437189811)
- dre:559564

- Down regulated genes

559564(-1.18025564761092)

- dre:140621

- Down regulated genes

140621(-2.68196234303566) 140621\_1(-3.95107590777093)

- dre:30762

- Up regulated genes

30762(1.51549567324805)
- dre:368878

- Up regulated genes

368878(2.50701188401484)
- dre:30763

- Up regulated genes

30763(5.58822372755117)
- dre:571282

- Up regulated genes

571282(1.90251155561911)

Close
